# Comprehensive Evaluation of 11 Cytokines in Premature Infants with Surgical Necrotizing Enterocolitis

**DOI:** 10.1371/journal.pone.0058720

**Published:** 2013-03-05

**Authors:** Thomas Benkoe, Suzann Baumann, Manfred Weninger, Mario Pones, Carlos Reck, Winfried Rebhandl, Rudolf Oehler

**Affiliations:** 1 Department of Pediatric Surgery, Medical University of Vienna, Vienna, Austria; 2 Department of Pediatrics, Division of Neonatology, Intensive Care and Neuropediatrics, Medical University of Vienna, Vienna, Austria; 3 Surgical Research Laboratories, Medical University of Vienna, Vienna, Austria; Universidad Pablo de Olavide, Centro Andaluz de Biología del Desarrollo-CSIC, Spain

## Abstract

**Objective:**

A prospective study to investigate the pattern of pro- and anti-inflammatory cytokine responses in neonates with surgical necrotizing enterocolitis (NEC) and identify those cytokines being the most promising for future research.

**Methods:**

A panel of 11 different cytokines were measured in 9 infants with proven NEC and compared with 18 age-matched healthy neonates.

**Results:**

The serum concentrations of the interleukins (IL)-6, IL-8, and IL-10 were significantly (32–fold to 56-fold) higher in NEC infants compared with controls. In contrast, IL-5, IFN gamma, IL-4 and IL-2 showed slightly (1.4-fold to 5.9-fold) lower levels in the NEC samples. However, these cytokines showed a very low absolute concentration in infants with NEC and in controls. The sum of the serum concentrations of IL-6, IL-8 and IL-10 was able to clearly separate infants with NEC from control samples. IL-1 beta and TNF-alpha showed no statistically different levels. The serum levels of TNF-beta and IL-12p70 were below the detection limit in more than 50% of all samples per group.

**Conclusion:**

In spite of strong local inflammation only three out of eleven cytokines (IL-6, IL-8, and IL-10) showed strongly increased serum levels indicating an important role of them in the pathogenesis of NEC. At least two of these three cytokines were elevated in every single NEC patient. Thus, longitudinal monitoring of combined IL-8, IL-6, and IL-10 levels could reveal their potency in being clinical relevant markers in NEC.

## Introduction

Necrotizing enterocolitis (NEC) remains one of the leading causes of morbidity and mortality in neonatal intensive care units, affecting up to 5% of premature infants [1]. Despite advances in the care of critically ill neonates, the mortality rates of NEC have remained unchanged in the past three decades ranging from 15% to 30% [2], [3]. In the group of infants with the most widespread disease, characterized by panintestinal involvement, the mortality rate approaches 100%. Surviving patients with NEC are facing significant morbidity, including neurodevelopmental impairment, feeding problems, failure to thrive, dependence on parental nutrition, and short bowel syndrome [4], [5]. Although the pathogenesis of NEC remains elusive, a deregulated inflammatory response by the neonatal intestine to luminal bacteria is a unifying hypothesis [6]. Intestinal epithelial injury is caused by different initiating events including intestinal ischemia, formula feeding, and colonization by opportunistic pathogens, leading to activation of the mucosal innate immune system and further damage of the epithelial barrier [7].

Animal models as well as several clinical observations indicate that the intestine of preterm infants react with an excessive inflammatory response to luminal microbial stimuli as compared to the adult intestine [6]. Correspondingly, NEC has been associated with increased levels of pro-inflammatory cytokines in the inflamed intestine itself as well as in the blood flow [8]. Animal models of NEC indicate that the release of pro-inflammatory cytokines promote intestinal ischemia and may under certain conditions cause septic shock in the absence of bacterial infection [9]. Accordingly, interleukin (IL)-1 alpha levels correlate highly to intestinal inflammation and necrosis in a rabbit colitis model [10]. Pre-treatment with the inhibitor IL-1ra reduced this inflammatory response. IL-6 neutralization in mice after intestinal infection with Yersinia, caused a dramatic decrease in local and circulating IL-1ra, suggesting that the pro-inflammatory IL-6 interplays with IL-1 indirectly via IL-1ra [11]. Anti-inflammatory cytokines are likely to play a protective role by inhibiting the inflammatory response. For example, intraperitoneal administration of IL-10 in an animal model of intestinal ischemia/reperfusion reduced local and systemic inflammation [12]. A recent study by Emami and co-workers [13] showed that IL-10 knockout mice developed more severe morphologic and histologic changes in a NEC model than wild type mice as evidenced by increased epithelial apoptosis, decreased junctional adhesion molecule-1 localization, and increased intestinal inducible nitric oxide synthase expression. Administration of exogenous IL-10 alleviated the mucosal injury. These animal model data show that different cytokines may have opposing effects. Thus, the course of the NEC disease depends finally on the relation of the different types of cytokines to each other. Such cytokine pattern studies have been extensively analyzed in adult patients with septic shock. But little is known about the ratio of pro-inflammatory to anti-inflammatory cytokines in preterm, very low birth weight infants suffering from NEC.

Elevated concentrations of the pro-inflammatory cytokine IL-6 have been reported in infants with NEC [14], [15]. In addition, several studies have detected elevated IL-8 protein in NEC specimens [16], [17]. IL-8 levels are significantly higher in infants with NEC compared to other inflammatory conditions [14]. We could recently show that the amount of IL-8 in the infant's sera seems to correlate with disease extent [18]. In addition, there are clinical studies reporting significantly elevated concentrations of the anti-inflammatory cytokine IL-10 in infants with NEC [15], [19]–[21]. The circumstance that all these study on different cytokines are measured in inconsistent study cohorts may withhold definitive conclusions which of the explored markers show the most promise for future clinical use [8]. A study by Lodha et al. [22] analyzed the blood levels of TNF alpha, IL-6 and IL-8 in NEC patients and compared them to growth and neurodevelopmental outcome. The results revealed that IL-6 and IL-8 levels elevated in the same patients while TNF alpha seemed to be completely independent. Unfortunately no anti-inflammatory cytokine has been investigated in this study.

To investigate the balance between pro-and anti-inflammatory cytokines we performed a prospective comprehensive evaluation of 11 key mediator cytokines in neonates with NEC.

## Materials and Methods

### Study design and population

This prospective study was conducted at the Medical University of Vienna following approval by the Ethics Committee of the Medical University of Vienna (Nr.875/2009). Informed written consent was obtained from parents of all study subjects. Infants with NEC undergoing surgical treatment were recruited during a 2 year time period from January 2010 to December 2011. Each of the NEC blood samples were obtained from infants exhibiting clinical signs and radiographic findings for proven NEC, according to modified Bell's staging criteria [23]. Only infants with stage II and stage III NEC were included into the study. The diagnosis of NEC was intra-operatively confirmed. NEC blood samples were obtained as part of the preoperative diagnostic workup within 6 hours prior to surgery.

Control blood samples were collected from infants with a birth weight of less than 2000 g, of less than 6 months of age, and with no clinical signs of infection. Physical signs of infection included at least 1 of the following: respiratory distress, feeding intolerance, abdominal distension, lethargy, irritability, or temperature instability. Further exclusion criteria included chronic inflammatory diseases and congenital malformations.

### Demographic and clinical parameters

Demographic parameters that were recorded and evaluated for all patients included birth weight, gestational age, 1-minute Apgar score, 5-minute Apgar score, and age at diagnosis of NEC. Important clinical data pertaining to NEC were also reviewed, including the presence of a patent ductus arteriosus, administration of ibuprofen, corticosteroids and antibiotics, use of mechanical ventilation, and application of vasopressors prior to diagnosing NEC.

### Flow cytometry

A Human Th1/Th2 PlexFlowCytomix kit (eBiosciences, Vienna, Austria) was used to quantify the serum cytokine levels. The assay was performed according to the manufacturer's instructions. Beads were run on a Gallios flow cytometer (Beckman-Coulter, Miami, FL, USA) for data collection. Further quantification was performed using the FlowCytomix Pro 2.2 software (eBiosciences, Vienna, Austria). The upper detection limit was for all cytokines 20,000 pg/ml. The lower detection limit differed between the cytokines (IL-12p70 27 pg/ml; IFN-gamma 15 pg/ml; IL-2 72 pg/ml; IL-10 2 pg/ml; IL-8 14 pg/ml; IL-6 1 pg/ml; IL-4 27 pg/ml; IL-5 5 pg/ml; IL-1beta 5 pg/ml; TNF-alpha 14 pg/ml; TNF-beta 2 pg/ml).

### Statistical analysis

We performed a study with 9 NEC subjects and 18 control subjects. Because there was no previous study investigating all different cytokines measured here, we based our power calculation on a previous study regarding IL-6 [22]. In that study the response within each subject group was normally distributed with a maximal standard deviation of 393 pg/ml. Based on these data we estimated the statistical power according to Dupont et al [24]. If the true difference in the experimental and control means is one standard deviation (393 pg/ml), we will be able to reject the null hypothesis that the population means of the experimental and control groups are equal with a probability (power) of 0.70. The Type I error probability associated with this test of this null hypothesis is p = 0.05 parameter. Some measurements gave results at or below the detection limit of the assay. Because this limit differed considerably between the various cytokines, we assigned the level of the detection limit instead of zero to these results. However, parameters showing results below the detection limit in more than 50% of all samples per group were treated differently. The statistical evaluation used in this study (Student's t-test) is based on the assumption that the data are normal distributed in each group. This cannot be assumed any more when more than half of the data of both groups have the same value. Such parameters were therefore rejected from the analysis. Hierarchical clustering was performed using SPSS software (ver. 18; IBM; Armonk, NY). The clustering was based on a median linkage analysis of the non-transformed serum levels using the Euclidean distance between cases or parameters. The color heat map was drawn using ECXEL 2010 software (Microsoft; Redmond, WA).

## Results

A total of 9 infants with surgically treated NEC were included and compared with 18 healthy infants as control group. Baseline perinatal characteristics including laboratory parameters in infants with NEC and age-matched healthy controls are summarized in Table 1. Clinical characteristics of infants with NEC recruited for cytokine analysis are displayed in Table 2.

**Table 1:** Neonatal characteristics of infants with NEC versus age matched controls.

	NEC (n = 9)	Control (n = 18)
**Male sex (n)**	6 (66%)	10 (%)
**Twins (n)**	4 (44%)	6 (%)
**Birth weight (g)** a	1200 (624–3200)	1130 (560–1994)
**Gestational age (weeks)** a	27 (24–40)	29 (24–33)
**Age at diagnosis (days)** a	24 (12–85)	28 (3–100)
**One-minute APGAR** a	8 (6–9)	8 (5–9)
**Five-minute APGAR** a	9 (8–10)	9 (7–10)
**Cases with 5-min Apgar <5 (n)**	0/9	0/18
**Intraventricular haemorrhage (n)**	6/9	4/18
**Patent ductus arteriosus (n)**	5/9	8/18
**Medical therapy of PDA (n)**	5/9	4/18
**Use of steroids (n)**	1/9	1/18
**Use of antibiotics (n)^b^**	6/9	0/18
**Mechanical ventilation (n)^b^**	4/9	0/18
**Use of vasopressors (n)^b^**	0/9	0/18
**Positive blood cultures (n)^b^**	1/9	0/18
**White blood cell count – K/mm3** a	5.66 (2.66–19.00)	10.52 (4.11–17.93)
**Platelet count – K/mm3** a	136.0 (13.0–345.0)	353.0 (115.0–622.0)
**CRP level – mg/dl** a	13.30 (0.72–21.65)	0.27 (0.10–0.60)

a median values; ^b^ at the time of diagnosis.

**Table 2:** Clinical characteristics of infants with surgical NEC recruited for cytokine analysis.

Patient no.	Sex	Gestational age, weeks	Birth weight, g	Apgar score 1 min	Apgar score 5 min	Postnatal age, days	Death
**1**	female	40	3200	8	9	17	No
**2**	male	27	1200	8	9	24	No
**3**	male	33	1550	9	10	12	No
**5**	male	27	1026	8	9	19	Yes
**6**	female	24	624	8	9	65	No
**7**	male	27	660	6	8	85	No
**8**	male	30	1600	8	9	14	No
**9**	female	25	1000	8	9	34	no
**10**	male	31	1490	7	8	26	no

Patient nr.4 did not receive surgical treatment and was excluded.

**Figure 1:**
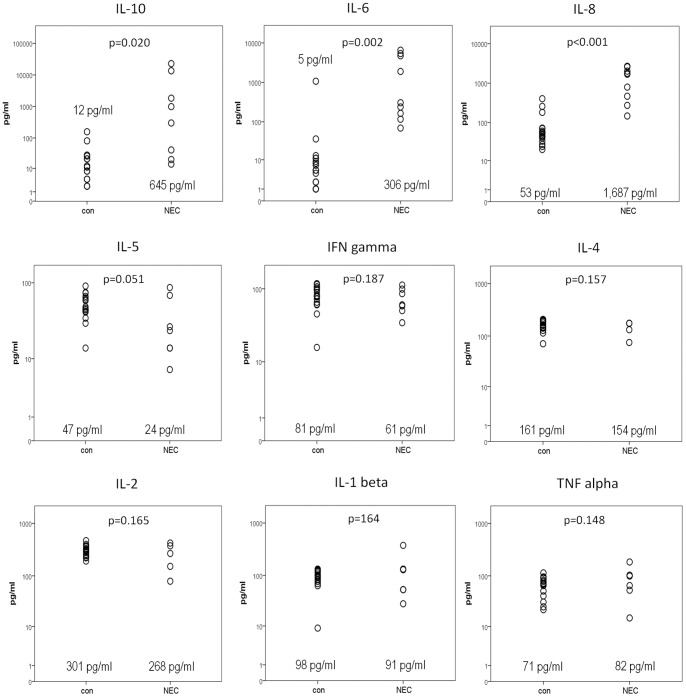
Serum concentrations of different interleukins in NEC patients (n = 9) and healthy age matched controls (n = 18). The median values of the respective group are indicated in the graph. Serum concentrations of Interleukin-12 (NEC and control group) and TNF-beta (NEC group) were below detection limit. The p-value was calculated using a Student's t-test.

Serum concentrations of Interleukin-12 and TNF-beta were below detection limit in more than 50% of all samples in both groups (NEC and control). They were therefore excluded from further analysis. Figure 1 indicates the concentrations of the remaining nine cytokines measured in the serum (a detailed list of individual cytokine levels is given in the Table S1). Cytokines IL-10, IL-6, and IL-8 were highly (38-fold, 56-fold, and 32-fold, respectively) increased in the NEC samples in comparison to healthy controls with only a few overlapping samples. In contrast, IL-2, IL-4, IL-5, and IFN-gamma showed slightly lower levels in the NEC samples than in healthy controls. However, the ratio was much lower (1.4-fold to 5.9-fold) and the overlap was much higher than in the previous group of cytokines. In addition, these cytokines showed a very low absolute concentration in both groups. IL-1 beta and TNF alpha showed no statistically different levels.

Calculating the correlation coefficients revealed that there is a clear correlation of IL-8 and IL-6 over all samples (NEC and controls; r = 0.88). Despite of a similar increase of serum IL-10 in NEC patients, it failed to correlate with IL-6 or IL-8 (r = 0.4 and 0.5, respectively). Some of the lowly expressed cytokines correlated with each other (r>0.7): IL-4 with IL-2, IL-5 and IFN-gamma; IL-2 with IFN-gamma and IL-5; TNF-alpha with IL-5 and IFN-gamma. However, because of the generally low serum levels of these cytokines we are not confident that these correlations are of physiological importance. Neither platelet count nor C-reactive protein correlated with any of the cytokines (r<0.7).

**Figure 2:**
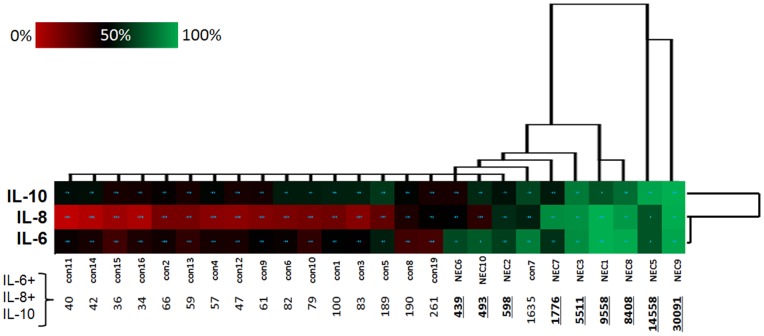
Heat map and unsupervised hierarchical clustering of 9 NEC patients and 18 healthy age matched controls according to the serum levels of IL-6, IL-8, and IL-10. The colored heat map indicates the relative serum level of the respective interleukin: red represents the 0 percentile, black the 50% percentile and green the 100% percentile. The clustering was based on a median linkage analysis of the non-transformed serum levels using the Euclidean distance between cases (vertical cluster) or parameters (horizontal cluster). The numbers beneath the heat map indicates the summation of the serum concentration of all three cytokines (in pg/ml).

In order to visualize serum levels of highly increased cytokines in the individual subjects a heat map was drawn (Figure 2). It shows that patients with high IL-10 levels (e.g. nec 3, nec 8, or nec 9) have also high levels of IL-8 and IL-6. Patient nec 5, in contrast, shows a high IL-10 concentration but intermediate levels of IL-6 and IL-8. A hierarchical clustering of the three cytokines revealed that IL-6 and IL-8 behaved much more similar than IL-10 (Figure 2, horizontal cluster). Only samples nec10, nec6 and nec7 showed clear differences between these two cytokines. Next we calculated an unsupervised hierarchical clustering over all samples (NEC and controls; Figure 2, vertical cluster). It shows that samples with high IL-10 or IL-8 levels (nec7, nec 3, nec 1, nec 8, nec 5, and nec 9) separate clearly from control samples. Samples nec 10, nec 6, and nec 2 cluster in the same group as the control. These latter samples, however, showed a high IL-6 concentration. Next we calculated the sum of the serum concentrations of IL-6, IL-8 and IL-10. Figure 2 shows that all NEC samples had a value above 300. Only one healthy control (con17) showed a higher value. Thus, considering all three cytokines improved the separation between NEC and controls in comparison to single cytokines.

## Discussion

The present study represents a comprehensive analysis of the serum cytokine pattern in surgical NEC patients and in age matched controls. In order to get an overview in the cytokine response to NEC, we used a cytokine array, which quantified the most relevant pro- and counter-inflammatory cytokines. This array would let us use very small samples of blood needed for concurrent cytokine analysis, an advantage given the limitations in patients with extremely low birth weight.

We found that only 3 out of 11 cytokines are strongly affected by the disease: IL-6, IL-8 and IL-10. Furthermore, we were able to demonstrate that all NEC samples have an elevated serum level of at least one of these three cytokines. Interleukin-6 is stimulated by a variety of pro-inflammatory cytokines, including TNF alpha and IL-1, and serves to activate lymphocytes and differentiation of cytotoxic T-cells [8]. Interestingly we could not confirm any NEC-associated elevation of TNF alpha or IL-1 levels. High levels of umbilical cord IL-6 were associated with neonatal disease processes like NEC and SIRS [25]. Our data corroborate reports by Harris et al. [26] and Romagnoli et al. [15] who reported a 5 to 30 fold increase of IL-6 levels in infants with NEC as compared with infants with sepsis and controls. Interleukin-8 is a potent pro-inflammatory chemokine, which has been already introduced in the pathogenesis of NEC. Several studies detected elevated IL-8 protein in NEC specimens [16], [17]. There is growing evidence that the amount of detected IL-8 in NEC is correlated with the degree of disease extent [14], [17]–[19]. Interleukin-10 has anti-inflammatory properties such as the suppression of in vitro synthesis of the pro-inflammatory cytokines TNF-alpha, IL-1, IL-6, and IL-8 [27], [28]. Moreover, IL-10 could play a critical role in modulating the mucosal pro-inflammatory response. Animal models have shown that IL-10 deficient knockout mice develop spontaneous enterocolitis [29] and that IL-10 deficiency exacerbates the degree of intestinal inflammation in response to a NEC inducing regimen [13]. In cases of clinical NEC, IL-10 levels were shown to increase in the serum of neonates [14], [15], [19], [21]. In line with previous data our study shows a significant up-regulation of IL-10 in infants with NEC, compared to controls.

Taken the results together, our data point to the important role of IL-10 in the pathogenesis of NEC and its relationship to the pro-inflammatory cytokines IL-6 and IL-8 in preterm neonates. Our results show that the up-regulation of IL-6 and IL-8 in NEC is accompanied by highly elevated IL-10 levels. As suggested in previous reports, we hypothesize that IL-10 levels in NEC display a compensatory mechanism to dampen the inflammatory response. Our data supports the hypothesis that the overwhelming activation of the pro-inflammatory cascade in NEC is not due to the lack of anti-inflammatory counter-regulation in NEC.

In the presented study we observed significantly lower levels of IL-2, IL-4, IL-5 and IFN-gamma in NEC samples compared with healthy controls. However, the ratio between infants with NEC and controls was much higher for the cytokines discussed above (Il-6: 38-fold, IL-6: 56-fold; IL-8 32-fold) than for these cytokines (IL-2: 3.8-fold; IL-4: 5.9-fold; IL-5 3.2-fold; IFN-gamma 1.4-fold). In addition, all these cytokines showed a much lower difference between infants with NEC and controls. Very little is known about the role of IL-2 and IL-5 in NEC. Interleukin-5 is involved in a number of immune responses such as asthma, helminth infection, and sepsis. Recently, Linch et al. [30] reported that the loss of IL-5 in sepsis resulted in increased mortality, IL-6 and IL-10 production, and bacterial burden. Furthermore, IL-5 is currently investigated as a cytokine based therapy in sepsis and asthma [30]. Chuang et al. [31] reported a small but significant increase of IL-5 secreting cells in the peripheral blood in infants with NEC. This is the first report on neonates with NEC and IL-2. The limited data on IL-5 and IL-2 in NEC warrants further investigation to clarify the role of these cytokines in the pathogenesis of NEC.

Interleukin-4 is a counter-regulatory cytokine, often defined as an immune modulator, as it inhibits the release of certain inflammatory cytokines, such as TNF alpha and IL-1b [32]. Studies in inflammatory bowel disease demonstrated an impaired IL-4 mediated down-regulation of IL-1b, TNF alpha, and therefore leading to uncontrolled pro-inflammatory cytokine release [33]. With regard to NEC this theory might be reasonable, as we observed significantly reduced IL-4 levels in infants with NEC, compared to controls.

It is of interest to note that we were not able to detect IL-12 and TNF beta in 50% of sera of neonates, neither in the NEC, nor in the control group. Although experimental NEC data indicated an increased number of IL-12 positive macrophages but no differences in the number of IFN gamma positive cells during the disease progression [34] we could not confirm these data in our clinical study. Increased IFN gamma has been demonstrated in neonatal rats and infants with NEC [7], [20]. But experimental studies showed that IFN gamma deficient mice still developed colitis when stressed [35]. It is questioned if these cytokines play a direct role in the development of NEC.

Very recently a study analyzed a NEC associated cytokine pattern using an antibody array assay to compare 174 different immunoregulatory molecules in 5 infants with NEC [21]. They found significantly at least 2-fold increased serum concentrations of IL-6, IL-8, and IL-10 in comparison to neonates with clinical features suggestive of gastrointestinal dysfunction. In addition, they could identify 20 other inflammatory mediators, which were not included in our analysis. In contrast to our work they demonstrated a two-fold increase in TNF beta. With the limitation of different control populations, our study showed similar results regarding IL-6, IL-8 and IL-10. The authors claim that the ratio of IL-6/IL-10 would be a valuable parameter to predict mortality of NEC patients, but failed to demonstrate a significant trend. In our hands, however, this ratio was not different between surgical NEC and healthy controls. This is because high IL-6 levels are accompanied by high IL-10 levels resulting in a reduction of fraction. All NEC patients of our study showed an elevated level of at least one of these three cytokines. Therefore, we propose to calculate the sum of IL-6, IL-8, and IL-10. We hypothesize that if Chan and colleagues [21] calculated their serum cytokine levels in the same fashion, they might have accomplished statistical significant results in predicting mortality.

In summary, the current study underlines the importance of IL-6 and IL-8 as pro-inflammatory cytokines in NEC. Moreover, the exaggerated release of IL-10 and the hypothesis that the overwhelming inflammatory reaction in neonates with NEC might not be linked to an impaired counter-inflammatory axis needs further clarification. We are aware that the study has its limitations. We did not include serial measurements of the evaluated cytokines in our study and therefore we are not able to display how cytokine levels respond to surgical treatment. Further studies involving larger patient series are needed before serum cytokine levels can be established as an unequivocally reliable parameter in clinical practice. However, our results provide insights for further investigations. The identification of clinical relevant inflammatory markers is crucial to make progress from bench to daily clinical practice. Longitudinal monitoring of IL-6, IL-8, and IL-10 could reveal a distinct cytokine pattern in dependence of disease severity. We assume that cytokine profiles obtained at the time when NEC is first diagnosed might be capable of distinguishing infants in need for surgery from prospective responders to medical treatment.

## Supporting Information

Table S1 **Cytokine levels of NEC patients and healthy controls.** The results are given in pg/ml (SD: standard deviation; p: significance level according to Students t-test).
(DOCX)
