# Supplementary material for: Comprehensive Evaluation of 11 Cytokines in Premature Infants with Surgical Necrotizing Enterocolitis
Source: PLoS One. 2013 Mar 5;8(3):e58720. doi: 10.1371/journal.pone.0058720 (PMC3589358; doi:10.1371/journal.pone.0058720)
Supplement: Table S1 — Cytokine levels of NEC patients and healthy controls. The results are given in pg/ml (SD: standard deviation; p: significance level according to Students t-test). (DOCX) [file pone.0058720.s001.docx]

| **SUPPLEMENTARY TABLE S1:** Cytokine levels of NEC patients and healthy controls. The results are given in pg/ml (SD: standard deviation; p: significance level according to Students t-test). | | | | | | | | | | | | | | | | | |  |
| --- | --- | --- | --- | --- | --- | --- | --- | --- | --- | --- | --- | --- | --- | --- | --- | --- | --- | --- |
|  | |  | |  | |  | |  | |  | |  | |  | |  | | |
| **Group** | **Patient no.** | | **IL-10** | | **IL-6** | **IL-8** | **IL-5** | | **IFN-γ** | | **IL-4** | **IL-2** | **IL-1β** | | **TNF-α** | | **IL8+ IL10+ IL6** | |
| NEC | 1 | | 300 | | 6.569 | 2.689 | 14 | | 99 | | 75 | 268 | 53 | | 65 | | 9.558 | |
| NEC | 2 | | 14 | | 116 | 467 | 7 | | 61 | | 132 | 152 | 53 | | 53 | | 598 | |
| NEC | 3 | | 1.838 | | 1.894 | 1.779 | 14 | | 51 | | 27 | 79 | 28 | | 14 | | 5.511 | |
| NEC | 5 | | 13.526 | | 238 | 794 | 24 | | 59 | | 27 | 72 | 374 | | 99 | | 14.558 | |
| NEC | 6 | | 2 | | 164 | 273 | 5 | | 15 | | 27 | 72 | 5 | | 14 | | 437 | |
| NEC | 7 | | 20 | | 69 | 1.687 | 27 | | 35 | | 27 | 72 | 5 | | 15 | | 1.776 | |
| NEC | 8 | | 990 | | 5.397 | 2.021 | 5 | | 15 | | 27 | 72 | 5 | | 14 | | 8.408 | |
| NEC | 9 | | 22.766 | | 4.737 | 2.588 | 87 | | 112 | | 175 | 423 | 132 | | 184 | | 30.091 | |
| NEC | 10 | | 41 | | 306 | 147 | 69 | | 86 | | 175 | 375 | 128 | | 103 | | 493 | |
| **median** |  | | **300** | | **306** | **1.687** | **14** | | **59** | | **27** | **79** | **53** | | **53** | | **5.511** | |
| SD |  | | 8.157 | | 2.653 | 985 | 30 | | 35 | | 66 | 143 | 118 | | 58 | | 9.662 | |
| Control | 1 | | 26 | | 13 | 60 | 91 | | 101 | | 198 | 399 | 132 | | 95 | | 100 | |
| Control | 2 | | 4 | | 7 | 55 | 47 | | 96 | | 202 | 306 | 121 | | 69 | | 66 | |
| Control | 3 | | 28 | | 11 | 44 | 75 | | 106 | | 205 | 315 | 118 | | 114 | | 83 | |
| Control | 4 | | 8 | | 5 | 43 | 61 | | 46 | | 145 | 278 | 76 | | 41 | | 57 | |
| Control | 5 | | 81 | | 36 | 72 | 64 | | 117 | | 192 | 297 | 111 | | 95 | | 189 | |
| Control | 6 | | 21 | | 9 | 51 | 74 | | 106 | | 208 | 470 | 128 | | 95 | | 82 | |
| Control | 7 | | 158 | | 1.072 | 404 | 45 | | 114 | | 178 | 375 | 102 | | 82 | | 1.635 | |
| Control | 8 | | 8 | | 1 | 181 | 45 | | 89 | | 159 | 258 | 96 | | 69 | | 190 | |
| Control | 9 | | 2 | | 11 | 47 | 67 | | 97 | | 189 | 319 | 125 | | 91 | | 61 | |
| Control | 10 | | 21 | | 2 | 56 | 59 | | 61 | | 141 | 358 | 82 | | 72 | | 79 | |
| Control | 11 | | 11 | | 8 | 20 | 45 | | 81 | | 164 | 226 | 84 | | 69 | | 40 | |
| Control | 12 | | 2 | | 5 | 40 | 49 | | 73 | | 145 | 221 | 69 | | 78 | | 45 | |
| conrtol | 13 | | 2 | | 2 | 55 | 42 | | 78 | | 149 | 192 | 100 | | 31 | | 57 | |
| conrtol | 14 | | 12 | | 5 | 24 | 35 | | 66 | | 145 | 333 | 90 | | 22 | | 42 | |
| conrtol | 15 | | 2 | | 1 | 33 | 30 | | 62 | | 114 | 243 | 63 | | 72 | | 34 | |
| conrtol | 16 | | 2 | | 4 | 27 | 14 | | 81 | | 128 | 301 | 92 | | 51 | | 34 | |
| conrtol | 19 | | 2 | | 1 | 258 | 5 | | 16 | | 71 | 72 | 9 | | 25 | | 258 | |
| conrtol | 20 | | 4 | | 1 | 70 | 43 | | 80 | | 164 | 297 | 100 | | 65 | | 75 | |
| **median** |  | | **8** | | **5** | **53** | **46** | | **81** | | **161** | **299** | **98** | | **71** | | **71** | |
| **SD** |  | | 39 | | 251 | 99 | 21 | | 26 | | 36 | 87 | 29 | | 26 | | 370 | |
| **p** |  | | 0.014 | | 0.001 | <0.001 | 0.021 | | 0.033 | | <0.001 | 0.007 | 0.146 | | 0.088 | | 0.001 | |
